# Integration Is Correlated With Mental Health Help-Seeking From the General Practitioner: Syrian Refugees' Preferences and Perceived Barriers

**DOI:** 10.3389/fpubh.2021.777582

**Published:** 2021-11-30

**Authors:** Samantha Marie Harris, Gro M. Sandal, Hege H. Bye, Lawrence A. Palinkas, Per-Einar Binder

**Affiliations:** ^1^Department of Psychosocial Science, Faculty of Psychology, University of Bergen, Bergen, Norway; ^2^Suzanne Dworak-Peck School of Social Work, University of Southern California, Los Angeles, CA, United States; ^3^Department of Clinical Psychology, Faculty of Psychology, University of Bergen, Bergen, Norway

**Keywords:** integration, help-seeking, mental disorders, Syrian, refugees, general practice

## Abstract

Despite a seemingly higher need, refugees in Europe tend to underuse mental health (MH) services. To better understand this underuse, it is important to understand refugees' willingness and ability to seek help from their general practitioner (GP) when experiencing MH problems. We employed a combined vignette and survey design to explore how the GP fits into the larger context of help-seeking preferences among a sample of Syrian refugees in Norway (*n* = 92), and what barriers they perceive in accessing help from the GP. We also examined how indicators of integration relate to seeking help from the GP. We take an exploratory approach. Participants were presented a vignette of an individual with symptoms in line with ICD-10 and DSM-5 criteria for depression. Participants were somewhat likely to seek help from the GP; however, seeking help from one's relationship with Allah/God and one's partner was preferred. Furthermore, while the GP was rated a somewhat likely help-seeking source, most participants indicated an average of two barriers to seeking help from the GP. Finally, social ties to the majority population in the form of social integration and feelings of connectedness with the host country (psychological integration) were positively correlated with likelihood of seeking help from the GP. Taken together, these findings suggest that the GP is considered a viable source of help among Syrians with a refugee background in the current sample, but that this may be influenced by perceived barriers and social as well as psychological integration. Addressing these barriers and promoting psychosocial integration with the host country are key to facilitating access and usage amongst refugees in need of MH services.

## Introduction

Despite a seemingly higher need for mental health (MH) support, refugees in Europe tend to underuse MH services (1). To better understand this underuse, it is important to better understand factors that influence refugees' willingness and ability to seek help from their general practitioner (GP) when experiencing MH problems. Throughout Europe, GPs are often the first line of contact for people seeking mental and physical healthcare (2, 3). GPs are trained to manage mild to moderate cases of MH problems (within primary care) and play a large role in determining appropriate preliminary diagnoses, assessments, treatments, and referrals for patients. Importantly, GPs are also often the first line of contact for patients with a refugee background.

One of the largest groups of refugees to arrive in Europe during the 2015–2016 refugee crisis originated from Syria. Syrian refugees who migrated to Europe and neighboring countries in the Middle East have reported experiences including civil war, torture, cultural integration issues, the loss of family and community support, discrimination and adverse political climate, loneliness and boredom, prohibition to work, and disruption of education for their children (4–6). Such stressors can place refugees at considerable risk of developing symptoms of depression, anxiety, post-traumatic stress disorder (PTSD) and related somatic health symptoms (7–12).

Considering MH and help-seeking in Syria may place refugees' engagement with health services in Europe into context. However, studies examining the MH burden in Syria are highly heterogeneous. A recent systematic review of the burden of mental disorders and access to MH and psychosocial support services in Syria and among Syrian refugees in neighboring countries found that levels of depression ranged from 11 to 49% (13). Similarly, another systematic review and meta-analysis recently estimated the prevalence of mental disorders at 21.1% in conflict-affected settings (14). However, mental healthcare in Syria has been neglected for decades (15), and studies on access and barriers to MH and psychosocial support in Syria are quite limited (13). It has been suggested that stigma associated with psychological and psychiatric disorders stands in the way of the use of MH services among Syrians in Syria (16, 17). Importantly, Syrians with a refugee background may seek help differently in Norway than Syria (17). This could relate to the different role of the GP in these countries. In Norway, patients, who want to see a specialist funded by the state are required to seek help via their GP (18). In Syria, however, patients are typically able to access specialist services directly, experiencing fewer delays and waiting times (17). Furthermore, the GP may be seen as a source of help for physical problems rather than MH problems. It is also worth noting that some services that are available in Norway, such as social workers, may not be a relevant source of help in Syria and may therefore not be considered.

Refugees' underuse of health services in high-income countries may, in part, be due to barriers to access and use. In Germany and Austria, Syrian refugees have identified barriers to help-seeking, such as stigma and shame, not speaking the language of the host country, and lacking information about health services (19–21). Similarly, refugees in Turkey have identified barriers such as not knowing where or how to get help, financial concerns, unavailability of appointments, fear of being hospitalized, and finding the process inconvenient or time-consuming (22). Such barriers may stand in the way of refugees accessing health care. The barriers perceived by Syrian refugees in a Norwegian context, however, have not been examined previously. The Norwegian public healthcare system is characterized by universal health coverage for all residents, although individuals make modest co-payments for different services. Services covered by universal health care include primary care, hospital care, and mental healthcare. Enrolment in universal healthcare is automatic, meaning that all residents have the right to state funded primary healthcare. Due to differences between countries' healthcare systems, we must assume that barriers perceived in other countries are not necessarily transferrable to the Norwegian context. Although it must be noted that refugees' expectations about the healthcare system in Norway may be influenced by their experiences in other countries.

Help-seeking is also influenced by factors besides barriers to accessing care. Andersen's behavioral model of health services use (BM) (23, 24) presents how contextual and individual factors, as well as health behaviors and outcomes, interact and influence the use of health care services (referred to as personal health services in the model). However, the experience of illness as well as preferences for seeking help are embedded in larger cultural and social systems (25). As an individual's cultural context changes, help-seeking preferences and behaviors are likely to change as well. This is supported by interviews with Syrian refugees (17). Similarly, preference for cultural traditions of the host country (including willingness to marry a Norwegian person, participating in social activities with Norwegians, etc.) was associated with semiformal (e.g., internet forum) and formal (i.e. medical doctor) as opposed to informal help-seeking sources among immigrants in Norway (26). This is in line with Wikberg and Eriksson (79), who claim that the more integrated an individual feels, the more likely they are to accept the host country's dominant care models. While previous studies have considered culture in relation to the BM (27), it may not suffice to include culture as a static variable, in the form of cultural values for example, without acknowledging the unique circumstances caused by shifting cultural contexts. This gap may be addressed by examining the concept of integration, defined as “the degree to which immigrants have the knowledge and capacity to build a successful, fulfilling life in the host society” (28, 29), in relation to help-seeking. Note, that the term “integration” does not imply that immigrants must surrender their own cultural identity and traditions to successfully integrate (30). Harder and colleagues (29) propose their multidimensional measure of immigrant integration spanning the domains of psychological, social, linguistic, economic, navigational, and political integration.

Harder's multidimensional measure of immigrant integration, or Immigration Policy Lab (IPL), can be used to measure integration overall or in its individual facets (linguistic, psychological, etc.), in contrast to several other measures, such as the Vancouver acculturation inventory (31) and the cultural competence scale (32–34) which examine similar facets, but combine these into an overall score. Furthermore, Harder's measure examines the current social situation of the participants, including amount of contact with members of the host society, while the Acculturation orientation scale (34) and Vancouver acculturation inventory (31) focus on individuals' preferences, including how important social contact with ingroups and outgroups is to participants. For the current study, we felt it was more helpful to employ a measure, which considers the participants' current situation rather than their preferences. Furthermore, as Harder et al. point out in the Supplementary Material of their paper, by “directly measuring the frequency of a social interaction, the question has face validity for measuring social integration” (29). Importantly, other scales assume that participants have friends in the resettlement country. Since this is not necessarily the case for all refugees, we have included single item measures to examine number of Norwegian and Syrian friends in Norway. Single item measures have been used previously to measure number of friends among refugee groups (35, 36). In a relatively hard to reach population, such as Syrian refugees in Norway, it is important that surveys remain short and concise. Harder's measure captures “key aspects of integration with a small number of widely applicable questions” and “can be used at low cost and facilitate comparability” (p. 11484). Their measure can therefore be used as a “common measure of integration, which would allow for the accumulation of knowledge through comparison across studies, countries, and time” [(29), p. 11483]. Harder's measure has furthermore been validated among relevant populations, including refugees as well as immigrants both in Europe and the United States (29).

The important role of integration in other domains besides help-seeking has been described previously, which lends support to the potential importance of this concept in help-seeking. A lack of social integration, for example, may be associated with decreased health-related quality of life, functional impairment, and severity of depression symptoms, anxiety, and PTSD (37). Similarly, while the beneficial impact of inter-ethnic friendships on the integration and well-being of migrant youths (38, 39) and adults (40) has been supported in previous research, the role of friendships in migration research has been largely treated as a side issue (41). Research conducted in Germany has suggested that social capital may facilitate integration of Syrian refugees into the labor market, and that different types of social capital may affect the outcome of the integration process (although the presence of social capital does not invariably lead to the successful utilization of that capital) (42). The importance of social integration has also been addressed by the German Institute for Economic Research, who suggest that social integration is vital in improving refugees' trust in key state institutions (43). Consequently, we incorporate elements of Harder's integration measure (2018) into Andersen's BM (2008; 2014) to guide the current study.

The present exploratory study focuses on the Norwegian context. While some specialist MH services exist for refugees, the majority that are officially settled in Norway are encouraged to contact their GP, who acts as a gatekeeper to specialist services (18). Like the rest of Europe, Syrian refugees in Norway have reported higher rates of MH problems. A recent cross-sectional study found that 33% of Syrian refugees in Norway reported symptoms indicative of anxiety or depression, and 7% reported symptoms of post-traumatic stress disorder (PTSD) (44). These rates are substantially higher than the 12-month prevalence of 10–15% for anxiety or depression (45), and 1–1.7% for PTSD (for men and women, respectively) among the Norwegian majority population (46). The aims of the current exploratory study are [1] to describe how the GP fits into the larger context of help-seeking preferences among Syrian refugees in Norway, and what barriers participants identify to accessing help from the GP, [2] to examine how the likelihood of seeking help from the GP relates to indicators of integration as well as other social, psychological, and demographic variables guided by the BM.

Furthermore, we focus on the GP as a source of help and the individual characteristics presented in the behavioral model (BM) as factors that may impact willingness to seek help from the GP. It is likely that throughout an individuals' life span either they or someone close to them will experience symptoms of depression. As a result, laypeople's beliefs about seeking help for depression have important implications for the behavior of people suffering from depression.

**Figure 1:**
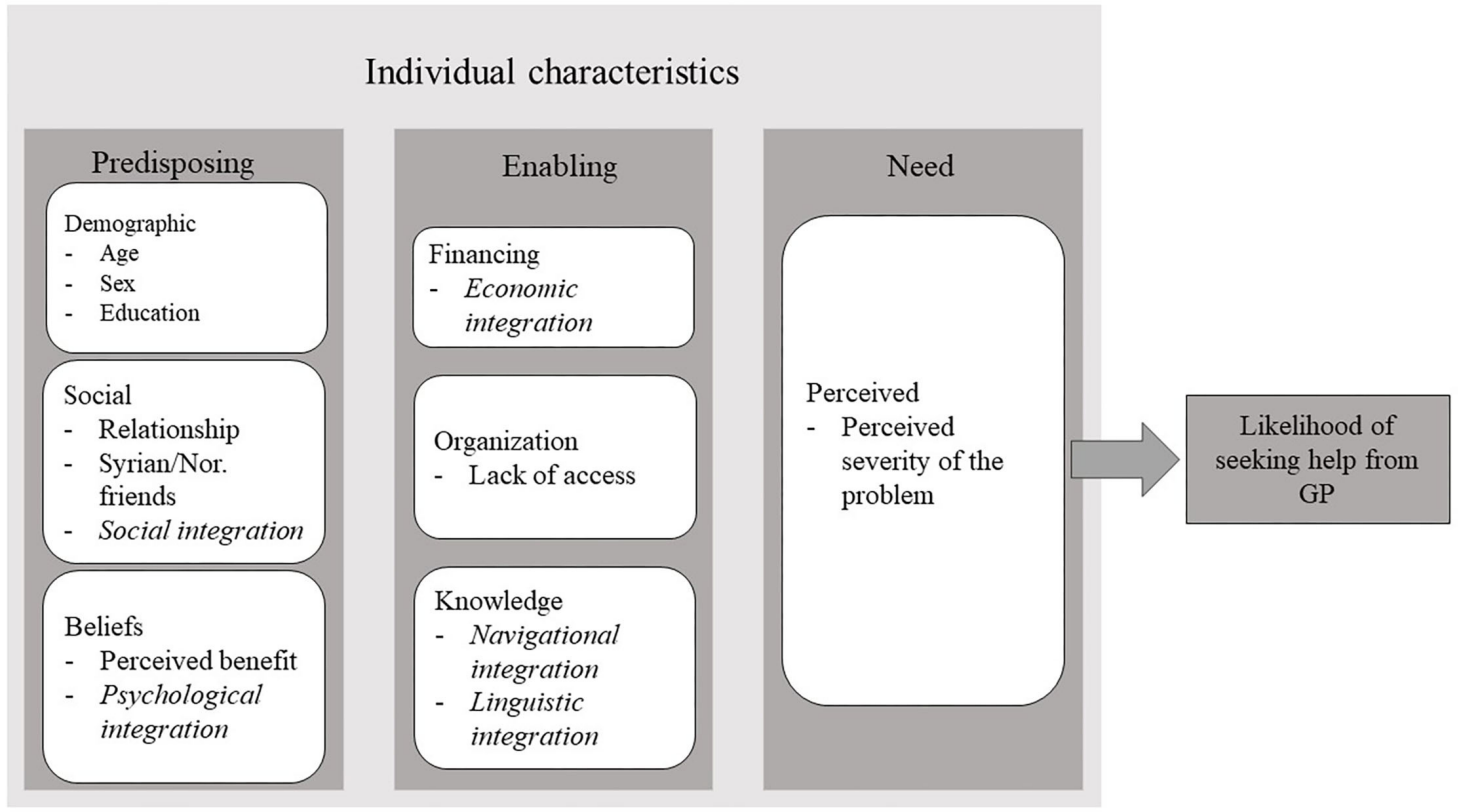
Conceptual model of the current study based on Andersen's behavioral model of health services use and Harder's multidimensional measure of immigrant integration (in italics).

According to the BM, the personal characteristics that influence help-seeking include predisposing (demographic, social, beliefs), enabling (financial resources, organization), and need (perceived and evaluated) (23, 24). While predisposing and enabling factors are sometimes difficult to disentangle (47), a predisposing factor can be thought of as something that would influence a person's willingness to seek help, while an enabling factor would influence their ability to receive help. Based on previous literature that has used the BM, we include age, gender, relationship status (47, 48), and education (48, 49) as predisposing demographic factors. In addition, we include perceived benefit of seeking help from the GP under predisposing beliefs, as this taps into the attitudes, values, and knowledge about health and health services (24). Here, we also incorporate Harder's social integration index and number Syrian and Norwegian friends, because this maps onto the social predisposing factors described in the BM (23, 24). We chose to include psychological integration under predisposing factors, as we felt it best related to beliefs about health and healthcare (23, 24), although it could also be argued that psychological integration can be seen as an enabling factor. We include Harder's economic integration index under enabling financing, similar to Johnson and colleagues (48). We examine lack of access to the GP under enabling characteristics, as it either facilitates or impedes health services use (23, 24), and does not predispose an individual to seek health help. We incorporate the indices for navigational and linguistic integration within a separate box entitled “Knowledge” within enabling factors, as these did not fit within another category of the model but have been shown to have important implications regarding help-seeking (1, 5, 10). Navigational integration, referring to an individual's ability to manage basic needs in the host country, best matched the element “organization” in the BM, which includes the existence of, and ability to access, a regular source of care. Furthermore, language proficiency, which is closely related to linguistic integration, has previously been included as an enabling factor (50). Finally, we include perceived severity of the problem under perceived need, because it captures participants' own perception of the severity of the symptoms. Severity of depression has previously been shown to relate to help-seeking (49), although it is unclear whether this is the case when responding to a vignette. Evaluated need from the original model is excluded as participants were not evaluated by a health professional. Our adapted conceptual model is presented in Figure 1.

## Materials and Methods

### Participants and Procedure

The current study was embedded in a larger survey study on refugees and MH. Our target population were Syrian refugees over the age of 18. We recruited participants through a purposive sampling strategy (51). Participants were mainly contacted through adult education programs^1^ in two large Norwegian cities. Most participants completed the survey onsite, either on their own mobile devices, or on an iPad provided by the researchers. Participants were also given the option to respond to a paper version of the questionnaire, and to complete the survey in Arabic or Norwegian. An Arabic speaking research assistant was available for support onsite. A link to the survey was furthermore advertised on the research group's official website and shared via personal and professional networks. Data were collected throughout 2019, and the final responses were collected on the 14th of February 2020. Recruitment of participants was planned to continue beyond this time frame but had to be terminated due to the COVID-19 pandemic and the ensuing lock down.

A total of 478 participants opened the survey link. Participants who consented to take part (*N* = 275) (57.5%) were randomized to one of two survey versions after answering demographic questions. Sixty-eight participants consented but dropped out prior to randomization. Of those that were randomized, 101 were randomized to the current study on help-seeking. Despite stating that we were recruiting participants from Syria with a refugee background, 4 individuals born in Norway participated. These were excluded from the final analysis. Similarly, participants were excluded if they did not respond to the help-seeking questions (*n* = 5), leaving a final sample of *n* = 92. Among these, there were some missing datapoints, but 82 completed the entire survey.

The final sample included 55 men and 37 women. Participants' ages were collected in 10-year age brackets. Most participants were between 30 and 39 years old (35.9%) followed by the 20–29 age group (37.0%). According to data from Statistics Norway (personal correspondence, 2021), of the 32,168 Syrians that moved to Norway between 2000 and 2021, 78% arrived as refugees and 22% arrived as family reunification cases. A vast majority of these individuals immigrated in 2015 and 2016. Consistent with this, we found that most of our participants indicated that their age of arrival corresponded to their present age group (58.7%) followed by having moved one age bracket up since arrival (39.1%). This suggests that our sample comprises recently settled refugees, in line with the pattern of immigration to Norway from Syria (52). Based on our sample size, (given α = 0.05, two-tailed), we had a power of 0.80 to detect a medium effect size of *r* = 0.31, a power of 0.99 to detect a medium to large effect of *r* = 0.45, and a power of 0.15 to detect a small effect size of *r* = 0.11 (53).

A minority of respondents reported being employed (21.7%), and most lived in a household with very low (33.7%) or extremely low (25.5%) annual incomes. The educational level of the respondents varied; many were educated at university/college level (51.1%), and about equal proportions of respondents indicated high school (14.1%) or elementary school (21.7%) as their highest completed level of education. Most of our sample were in a relationship (married or cohabiting) (64.1%) and about half (46.7%) had children. Participants' demographic characteristics are presented in Supplementary Table 1 in the Supplementary Material.

Given the high prevalence of depressive symptoms and related MH problems in refugee populations, it is likely that some of our participants experienced depressive symptoms at the time of the survey. We included common psychiatric disorders, general self-rated health, and identification with the vignette character in the current study to examine the relationship between these variables and what participants report they would do in case they felt like the vignette character.

### Measures

#### Help-Seeking

To measure help-seeking preferences, participants were presented with a vignette describing an individual, who was experiencing symptoms in line with DSM-V and ICD-10 criteria of depression (54, 55). The vignette is the same as used by Aarethun et al. (17), Markova et al. (56), and Markova et al. (26), which is based on Erdal and colleagues (57). Female participants were presented with a female vignette character and males with a male vignette character. The vignettes were otherwise identical (Supplementary Material 2).

After reading the vignette, participants indicated how likely they were to seek help from different sources, if they felt like the vignette character (6-point Likert scale where 1 = Very unlikely, 6 = Very likely, and 7 = NA). Participants could select from a list of different sources, based on categories used by Markova et al. (26) and the General Help-seeking Questionnaire (58). Next, the participants were asked to indicate their first, second, and third most preferred help-seeking sources.

#### Barriers to Seeking Help From the GP

Based on barriers commonly mentioned in the literature (59–62) we developed a list of potential barriers for seeking help from the GP. Similar barriers have since been described in more recent studies (1, 63). The list of possible barriers is presented in the results section.

#### Integration Indices

We employed the integration indices as described in the Supplementary Material of Harder et al. (29). We followed the IPL-12 (Immigration Policy Lab-12) version of the measure for all indices apart from social and psychological integration, for which we included additional items from the IPL-24. Note that we excluded the index for political integration, as it had no clear link to help-seeking preferences.

#### Social Integration

The social integration index consisted of three items, such as “In the last 12 months, how often did you eat dinner with *Norwegians* who are not part of your family?” (1 = Never, 5 = Almost every day). The index had “acceptable” internal consistency (α = 0.64) according to previous literature (64).

#### Psychological Integration

The psychological integration index consisted of four items, such as “How connected do you feel with Norway?” (5 = I feel an extremely close connection, 1 = I do not feel a connection at all). The index had good internal con sistency (α = 0.83).

#### Linguistic Integration

Linguistic integration was measured by two items as follows: “Communicating in *Norwegian* has many components, like reading, writing, and speaking skills. Please evaluate your own skills in *Norwegian*”: “I can read and understand the main points in simple newspaper articles on familiar subjects” and “In a conversation, I can speak about familiar topics and express personal opinions” (5 = Very well, 1 = Not well at all) (*r* = 0.83).

#### Navigational Integration

We initially based navigational integration on the two items included in the IPL-12 (29): “In this country, how difficult or easy would it be for you to do each of the following? (A) See a doctor. (B) Search for a job (find proper listings)” (1 = Very difficult, 5 = Very easy). However, the items were uncorrelated in our sample (*r* = 0.07). Therefore, we employ only the single item regarding finding a doctor, which was most relevant to the scope of this paper.

#### Economic Integration

The economic integration index used in the current study consists of one item examining household income equalized by household size. Originally, this item is to be combined with occupational status, but these two items were uncorrelated in our sample (*r* = 0.09), and we thus focus on equalized household income only.

#### Number of Norwegian and Syrian Friends

Number of Norwegian and Syrian friends was examined through the items “Do you have one or more Norwegian friends” and “Do you have one or more Syrian friends?” (1 = No, 2 = Yes, I have one friend, 3 = Yes, I have several), which was dichotomized for the analysis (1 = No, 2 = Yes, I have one or several friends).

#### Perceived Severity

Perceived severity was measured by asking participants whether they felt the vignette character's condition was severe enough to warrant sick leave (Yes/No).

#### Identification With the Vignette Character

We measured identification with the vignette character by asking participants to what extent two progressively overlapping circles represent them and the vignette character. Circles A, for example, represented two separate circles (coded as 1), while circles G were almost entirely overlapping (coded as 7).

#### Self-Rated Health

Participants' general self-reported health (GSRH) was measured through the single item: “Overall, would you say your health is:” with the response options ranging from (5) Excellent to (1) Very Poor. This question has previously been used to measure self-rated health among Syrian refugees migrating to Norway (65) and has been validated among Arabic speaking refugee populations (66).

#### Common Mental Disorders

Common mental disorders were measured using the HSCL-25 (67). Participants were asked to report to what extent a range of experiences applied to them over the last 14 days (1 = Not at all, 4 = A lot). The Norwegian and Arabic translations of this survey have been validated in Norwegian and Arabic samples (68, 69). In our sample, mean HSCL score for men was 2.20 (SD = 0.71) and 2.04 (SD = 0.67) for women. Of these, 63% of women and 75% of men scored above the clinical cut-off of 1.75 (70). While we are cautious to determine an optimal clinical cut-off in the current sample, it appears that a substantial number of participants reported symptoms indicative of psychological distress.

### Ethical Considerations

This study was approved by the Norwegian Center for Research Data (NSD Notification form: 602214). All participants gave written consent in accordance with the Declaration of Helsinki (71) at the start of the survey. Participation was voluntary, anonymous, and confidential.

## Results

### The GP as a Source of Help

Participants' likelihood of seeking help from different sources is presented in Table 1. The GP ranked as the fourth most likely source of help, preceded by Allah/God, participants' partner, and mother. To further explore the likelihood of seeking help from the GP in comparison to other positively rated help-seeking sources, we conducted a series of paired samples Wilcoxon signed rank tests. Due to the number of tests and the exploratory nature of our analyses, we employed a more stringent alpha level of 0.01. These tests revealed that the likelihood score of seeking help from the GP was significantly lower than that of seeking support from Allah/God (*T* = 5453.5, *p* < 0.001) and one's partner (*T* = 2721.5, *p* = 0.009). However, we found no significant difference between the mean likelihood rating of seeking help from the GP and one's mother (*T*= 3578.5, *p* = 0.530), a psychologist/psychiatrist (*T* = 4132, *p* = 0.811), other family members (*T* = 4209.5, *p* = 0.460), Syrian friends (*T* = 4322.5, *p* = 0.124), father (*T* = 3306, *p* = 0.279), the internet (*T* = 4870, *p* = 0.011), or Norwegian friends (*T* = 4707, *p* = 0.012). The likelihood score of seeking help from the GP was significantly higher than seeking help from a social worker (*T* = 5038.5, *p* ≤ 0.001).

**Table 1:** Participants' likelihood of seeking help from different sources ordered by highest (top) to lowest (bottom) total mean likelihood score, standard deviation (SD), median, and interquartile range (IQR).

		**Total**	
**Help-seeking source**	***N***	**Mean (SD)**	**Median (IQR)**
Allah/God	86	5.13 (1.56)	6 (5–6)
Partner	79	4.56 (1.82)	5 (4–6)
Mother	85	4.05 (1.94)	5 (2–6)
GP	89	3.98 (1.76)	4 (2–6)
Psychologist/Psychiatrist	91	3.88 (1.84)	4(2–5)
Other family member	89	3.80 (1.77)	4 (2–5)
Syrian friends	86	3.55 (1.81)	4 (2–5)
Father	82	3.54 (2.09)	4 (1–6)
Internet	90	3.33 (1.68)	4 (2–5)
Norwegian friends	87	3.30 (1.74)	4 (2–5)
Social worker/NAV	89	3.08 (1.80)	3 (1–5)
Nurse	91	3.01 (1.64)	3 (2–4)
Physiotherapist	86	2.85 (1.73)	2 (1–5)
Elders in my community	88	2.84 (1.60)	3 (1–4)
None	89	2.75 (1.77)	2 (1–4)
Colleague/someone at work	90	2.74 (1.65)	2 (1–4)
Teacher/contact from introductory programme	90	2.71(1.65)	2 (1–4)
Telephone helpline	81	2.53 (1.56)	2 (1–4)
A&E	88	2.52 (1.67)	2 (1–4)
Alternative treatment*	85	2.42 (1.68)	2 (1–4)
Other	82	2.39 (1.71)	2 (1–3)
Charity	86	2.38 (1.57)	2 (1–3)
Religious leader (e.g., imam or priest)	88	2.33 (1.59)	2 (1–4)
Traditional healer from my country of origin	86	2.05 (1.35)	2 (1–3)
Leader from my community or country of origin	86	1.81 (1.12)	1 (1–2)

Responses were indicated on a 6-point Likert scale, where 1 = very unlikely and 6 = very likely, 7 = NA. Responses are based on a sample size of N = 92. N in the table presents participants who chose a likelihood rating between 1 and 6. Not all sources of help may have been relevant for all participants. *Alternative treatment was specified as: e.g., acupuncture, homeopathy, herbal treatment.

**Figure 2:**
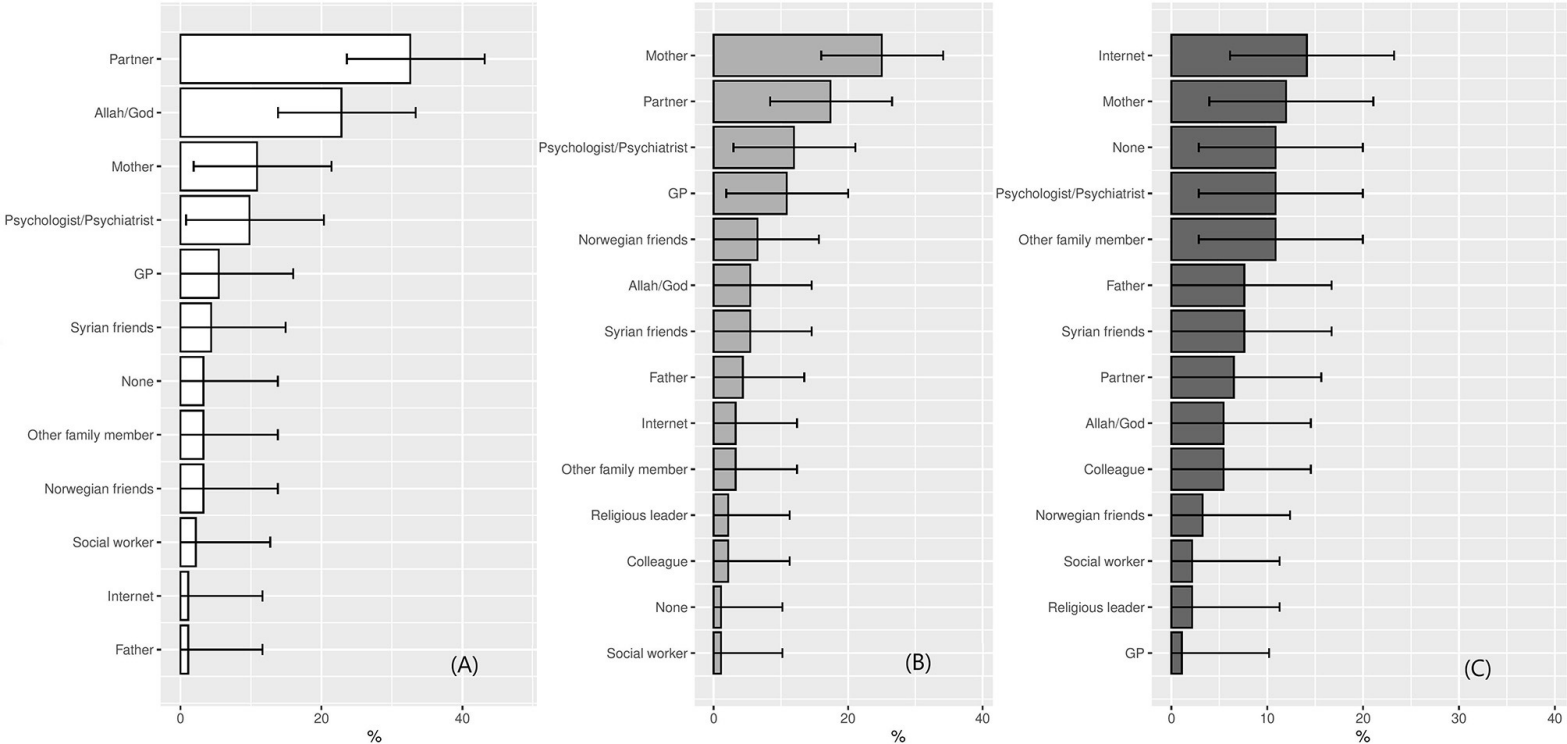
**(A–C)** Help-seeking choices (*N* = 92). Note that none of the participants chose “religious leader” and “colleague” as their first choice. The following help-seeking options were removed from the figure, as they were not chosen by participants at all: Helpline, A&E, Traditional healer, Elder, Alternative healer, Leader from community, Teacher, Nurse, Charity, Physiotherapist, and Other.

Figure 2 presents participants' top three help-seeking choices, based on the question “where would you seek help first, second, or third.” The most frequent first choices were partner, Allah/God, and mother. The most frequent second choices were mother, partner, and psychologist/psychiatrist followed closely by the GP. Finally, the third choices were spread more evenly, with the internet ranking as the most common third choice, followed closely by mother and none, which was chosen similarly as often as psychologist and other family member. Figure 2 extends the findings from the paired Wilcoxon rank tests and suggests that there is a preference for seeking help from Allah/God and one's partner over seeking help from one's GP.

### Barriers to Seeking Help From the GP

**Figure 3:**
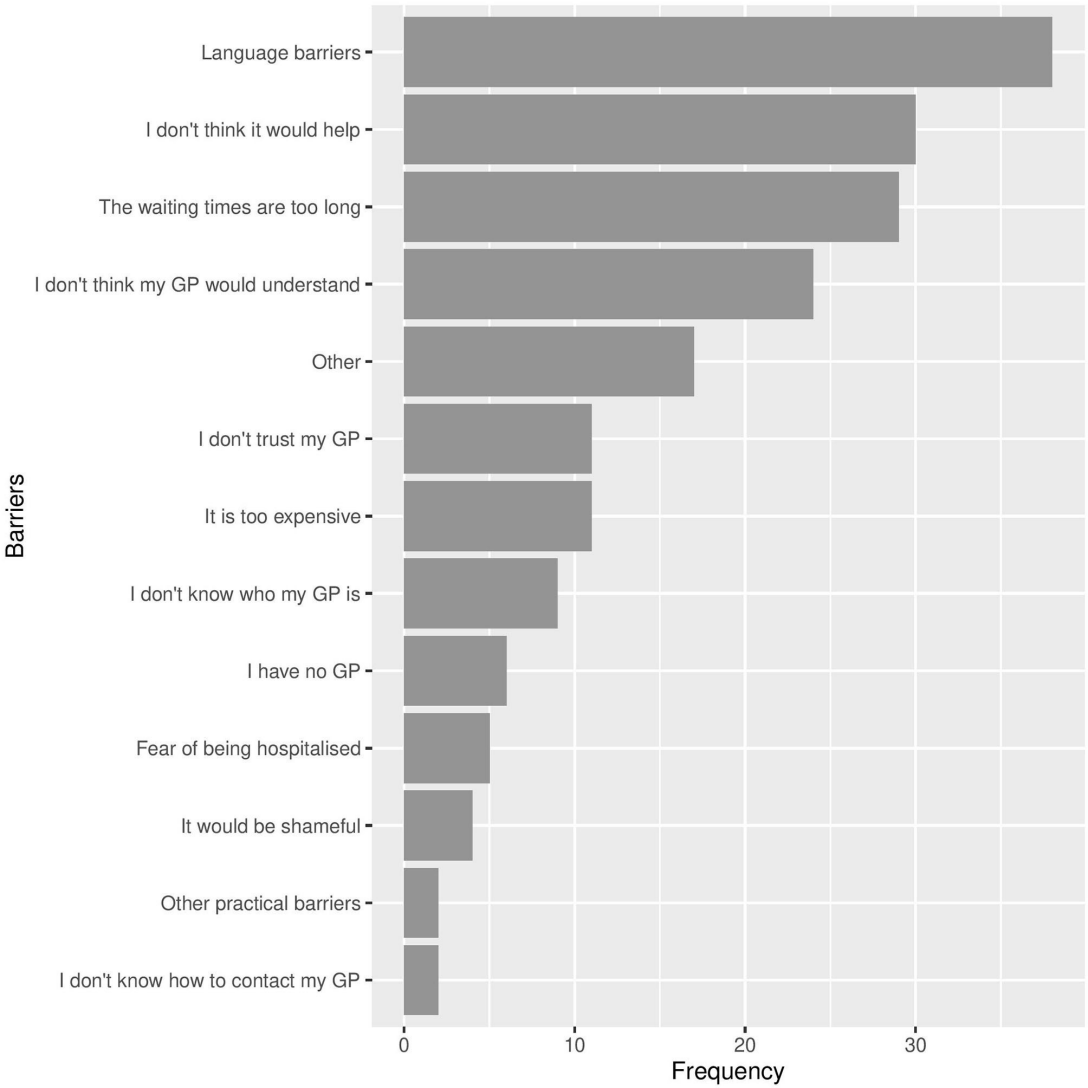
Barriers reported by participants to seeking help from the GP (*N* = 87). Note that participants could select several or no barriers.

Barriers to seeking help from the GP are presented in Figure 3. Of 92 participants, 87 identified at least 1 barrier. On average, participants reported 2 barriers (*SD* = 1.8). The most frequently chosen barriers were “language barriers,” “I don't think it would help,” “the waiting times are too long,” and “I don't think my GP would understand”.

We created a measure of lack of perceived benefit by combining the barriers “I don't think it would help,” “I don't think my GP would understand,” and “I don't trust my GP.” Lack of perceived benefit of seeking help from the GP was negatively correlated with likelihood of seeking help from the GP, [*r*_*s*_(87) = −0.35, *p* < 0.001]. We did not find, however, lack of access in the form of not having a GP, not knowing who the GP is or how to contact them, to be central barriers in our sample. The final element of access, waiting times—endorsed by 27 of 87 participants –, was positively correlated with seeking help from the GP [*r*_*s*_(87) = 0.22, *p* = 0.038]. The fact that individuals had the experience of long waiting times suggests that they had access to their GP, and the positive correlation indicates that long waiting times did not systematically deter participants from considering the GP as a viable source of help. Given the emphasis placed on stigma and shame in previous research, it is notable that very few participants (*n* = 3) indicated that seeking help form the GP would be shameful.

### The Role of Integration in the Behavioral Model

Our second aim was to examine several socio-demographic variables based on previous literature and their relation to endorsing seeking help from the GP, as well as address integration's role in the model. Correlations between all variables are presented in Table 2. In terms of socio-demographic variables, we found that neither gender nor education were related to endorsing help-seeking from the GP.

**Table 2:** Spearman's rank (in gray) and pearson correlations among study variables.

	**1**.	**2**.	**3**.	**4**.	**5**.	**6**.	**7**.	**8**.	**9**.	**10**.	**11**.	**12**.	**13**.	**14**.	**15**.	**16**.
1. Seeking help from GP	–															
2. Gender	−0.02	–														
3. Age	0.27^*^	−0.16	–													
4. Education	0.05	−0.10	0.03	–												
5. Relationship	−0.26^*^	−0.17	0.01	0.01	–											
6. Syrian friends	0.11	−0.05	−0.04	0.13	−0.03	–										
7. Norwegian friends	0.09	0.03	0.23^*^	−0.04	−0.25^*^	0.07	–									
8. Lack of benefit	−0.35^*^	−0.06	−0.17	0.07	0.02	−0.06	−0.19	–								
9. Severity	−0.02	0.18	0.00	0.00	−0.10	0.10	0.10	−0.15	–							
10. Vignette character identification	−0.05	0.04	−0.16	−0.23^*^	0.13	0.00	−0.09	0.04	−0.08	–						
11. Health	−0.15	0.01	−0.15	0.05	−0.11	−0.02	0.27^*^	−0.06	0.19	−0.34^*^	–					
12. HSCL	−0.03	−0.11	−0.16	0.03	0.16	0.00	−0.25^*^	0.30^*^	−0.16	0.57^**^	−0.51^**^	–				
13. Social integration	0.32^**^	−0.10	0.28^*^	0.03	−0.23^*^	0.13	0.56^**^	−0.20	0.06	−0.08	0.20	−0.15	–			
14. Psychological integration	0.24^*^	0.05	0.12	−0.21	−0.15	−0.22^*^	0.27^*^	−0.20	−0.02	−0.18	0.26^*^	−0.35^*^	0.34^*^	–		
15. Linguistic integration	−0.07	−0.16	−0.06	0.28^*^	−0.05	0.01	0.07	0.19	−0.06	−0.01	−0.06	0.06	0.12	−0.09	–	
16. Economic integration	−0.06	−0.14	0.01	0.19	0.00	−0.03	−0.01	0.01	−0.07	0.04	−0.09	0.10	0.10	0.07	0.27^*^	–
17. Navigational integration	0.07	−0.16	0.04	0.06	0.00	−0.10	0.10	−0.18	0.14	−0.25^*^	0.28^*^	−0.30^*^	0.01	0.21	0.18	0.05

*Note. Gender (1 = male, 2 = female), Relationship (1 = Yes, 2 = No), Syrian/Norwegian friends (1 = No, 2 = Yes, I have one or several)*.

* *p < 0.05,*

** *p < 0.01*.

Higher psychological [*r*_*s*_(83) = 0.24, *p* = 0.028] and social integration [*r*_*s*_(81) = 0.32, *p* = 0.003] were both positively correlated with likelihood of seeking help from the GP. We did not find any significant associations between having Norwegian friends, Syrian friends, economic, linguistic, or navigational integration and reported likelihood of seeking help from the GP. It is worth noting, however, that while there was no significant correlation between number of Norwegian friends and likelihood of seeking help from the GP, both psychological and social integration were significantly correlated with Norwegian friends (see Table 2), suggesting that the effect of Norwegian friends may be indirect.

### Perceptions of Severity and Participants' Own Health Status

Neither perceived severity of the condition [*r*_*s*_(87) = −0.02, *p* = 0.875], participants' own self-reported health status [*r*_*s*_(83) = −0.15, *p* = 0.157], mean HSCL score [*r*_*s*_(81) = −0.03, *p* = 0.818], nor their identification with the vignette character [*r*_*s*_(86) = −0.05, *p* = 0.651] was correlated with endorsing seeking help from the GP. While we did not include these variables in the conceptual model, these results suggest that individuals are not influenced by their current health status when considering potential sources of help for the future. Furthermore, these variables act as validity checks, in that HSCL score is correlated with identification with the vignette character [*r*(84) = 0.57, *p* < 0.001], as well as self-reported health [*r*(84) = −0.52, *p* < 0.001].

## Discussion

### Summary of Results

The findings of the current exploratory study suggest that Syrians with a refugee background considered seeking help from the GP as somewhat likely if they experienced symptoms in line with depression. Formal sources of help, such as the GP and psychologist/psychiatrist, were preceded by Allah/God and one's partner as preferred sources of help. Furthermore, participants indicated experiencing an average of two barriers to seeking help from the GP. The most prevalent barriers included language barriers, not thinking it would help, long waiting times, and feeling like the GP would not understand. We found that psychological and social integration, i.e., feelings of connectedness with Norway and having a Norwegian social network, were correlated with higher reported likelihood of endorsing the GP as a viable source of help.

### Previous Literature and Implications

#### The Role of the GP as a Source of Help

Our findings suggest that Syrians with a refugee background in the current sample preferred to seek help from Allah/God and their partner over the GP or psychologist/psychiatrist, but that the GP and psychologist/psychiatrist were nevertheless considered viable help-seeking sources. This has been found among Syrian refugees in Istanbul, who reported a preference for seeking help from informal sources, such as family, but also endorsed seeking help from professional sources (22). Participants in the latter sample also reported religious leaders as a common source of help (22), while our sample ranked religious leaders as very unlikely help-seeking sources and Allah/God as a very likely source of help. Our findings, therefore, suggest there may be an important difference between turning to one's relationship with Allah/God for help and seeking help from religious leaders. We must also consider that some sources of help, such as social workers, may not be relevant in Syria, which may explain why participants considered social workers as a relatively unlikely source of help in the current study. Nevertheless, all refugees receive information about MH and formal help-seeking sources as part of the introductory program, which would have raised awareness about social workers and their role in Norwegian society.

It is important to remember that help-seeking sources are not mutually exclusive (5), and individuals may consider seeking help from both formal as well as informal sources simultaneously. Adopting both dominant care models as well as the care models of the home country has the advantage of affording individuals more options (72). However, Atallah (72) draws particular attention to the conflict that may arise when the dominant care model of the patient's home and host country do not align. The current study finds no evidence for a conflict on the side of the participants, but we must acknowledge that a conflict may exist on the side of the practitioner. Practitioners in Norway are not expected to consider or recommend religious coping, despite evidence that religious coping has a range of benefits for mental well-being (73).

#### Barriers to Seeking Help

Commonly identified barriers to help-seeking, like language and waiting times (19–22), are in line with our results. Our findings also mirror reports from GPs working with refugee patients with MH problems (74). Language barriers in particular have been identified by GPs as obstacles to providing mental healthcare to refugees (74). Similarly, GPs have reported feeling as though refugee patients had different understandings of what constitutes and causes health and illness, resulting in a lack of understanding one another (74). The barrier “I don't think my GP would understand” chosen by participants in the current study mirrors this experience and suggests that language barriers as well as different understandings of health and illness may be perceived by both patients with a refugee background and their GPs.

Lack of perceived benefit was also indicated as an important barrier by our sample. Perceived benefit has previously been identified as one of the most important predictors of help-seeking intentions among adolescents (75). High perceived benefit may lead other barriers to become less important, and if confirmed in future research, may suggest that health promotion programmes, which focus on removing barriers should also promote the benefits of seeking help for MH problems (75).

However, other common barriers identified previously, such as stigma, shame, not knowing how to contact the GP, financial concerns, and fear of being hospitalized were not mirrored in our findings (19–22). With regards to shame and stigma, focus group interviews with Syrian refugees in Norway suggest that while stigma and shame influenced where individuals might seek help, they also acknowledged that stigma surrounding professional healthcare was diminished in Norway, making it easier and less stigmatized to seek professional MH help (17).

Finally, our findings suggest that current health status is not associated with considering the GP as a source of help; however, our findings do suggest that current health status is associated with other forms of help-seeking behavior^2^. This is in line with previous literature, which suggests that severity and duration of depression as well as chronic somatic disorders are related to help-seeking behaviors (49).

#### Role of Social Networks and Feelings of Connectedness

Intergroup contact and feelings of connectedness with the host country have previously been found to relate to well-being among refugees (37, 76). Less attention has been paid to psychological integration. Our results extend previous findings and address this gap in the literature, suggesting that social ties and feelings of connectedness, measured through the social and psychological integration indices (29), play an important role in considering seeking MH help from the GP. This further ties in with the importance of social capital, which has been shown to play a role in other domains, such as gaining access to the job market (42). Gericke et al. (42) distinguish between different types of social capital: bridging, referring to social contact with individuals outside of your community, vs. bonding social capital, marked by closed-off communities. They suggest that bonding social capital may put individuals at a disadvantage regarding accessing career-related information and social mobility. Furthermore, the authors highlight the difference between horizontal social capital, between people with similar access to resources and knowledge, and vertical social capital, which describes contacts who belong to different social levels (42). By the same virtue, Syrians with a refugee background in the context of the current study may benefit from having close ties to the Norwegian majority population, i.e., vertical, bridging social capital, with regard to the help-seeking sources they consider, and, consequently, are afforded more options regarding where to seek help. However, it is important to note that our sample had little variation regarding financial and employment situation. Consequently, our findings suggest that within an economically relatively deprived sample, social, and psychological integration play an important role in participants' consideration of professional sources of help, and do not necessarily imply that economic, navigational, or linguistic integration are not also important factors.

### Strengths and Limitations

This study makes an important contribution by recognizing that help-seeking preferences are dynamic and contingent on time and context, rather a static characteristic. Similarly, much of the previous literature has framed help-seeking among refugee and non-refugee migrants in terms of barriers and factors that put them at a disadvantage to majority populations. While we also present barriers, our findings highlight social ties and feelings of connectedness as facilitators to help-seeking. Having collected data from Syrian refugees, our findings are particularly relevant to this patient group. Refugees are a highly heterogeneous group and “lumping them together” is neither appropriate nor informative (77). However, it should be noted that by the same token our findings may be less relevant for other patient groups. This may also be the case for important intragroup heterogeneity. For example, we did not consider ethnic identification within this participant group. Disregarding the differential culture of Kurds, for example, can gloss over important cultural differences that may play a role in help-seeking. Next, given the cultural differences presumed to exist with regard to our understandings of mental illness (5), we chose to present a vignette, which did not mention the term depression but instead described only the symptoms. This allowed us to gain an insight into individuals' help-seeking preferences for such symptoms without entangling our study in a larger discussion around the cross-cultural validity of “Western” nosology. The use of this vignette furthermore allowed Aarethun et al. (17) findings to complement the findings of the current study.

The study also had certain limitations that suggest caution should be exercised when interpreting the findings. We examined help-seeking preferences of Syrians with a refugee background regarding a fictional vignette character, which may not reflect participants' true help-seeking behaviors. Nevertheless, the preferences indicated in the current study may present what individuals are likely to endorse in situations where family and friends seek advice from them. Given the importance of certain informal help-seeking sources among this sample, this information is highly relevant. It is also important to note that help-seeking may differ by migrant background. Quota refugees and family reunification refugees in Norway are screened by a doctor, where many cases of MH problems are identified and managed. We chose not to collect information regarding specific reasons for arrival, as we felt this was too intrusive. However, if participants were to a large extent quota or family reunification refugees, it is possible that many are familiar with the Norwegian healthcare system. This may have impacted their willingness to consider the GP as a viable source of help, and therefore influenced our results. Similarly, we did not collect specific data regarding time spent in Norway, which has been shown to be an important variable regarding help-seeking in previous literature (1). Time spent in the host country is often employed as a proxy for integration or acculturation. The use of proxies, however, may be imprecise and implies that integration and other relevant processes progress similarly for all migrants over time. Our study improves this approach by examining integration directly. It is, however, important to note that we employed several single item measures. Both navigational and economic integration were intended to be measured through several items, but due to a lack of correlation between items we reduced these to single measure items. This is sample specific and should be corrected in future research. Finally, the social integration measure had a lower than desired reliability in the current sample. Nevertheless, the measure correlated meaningfully with other variables, such as seeking help from the GP, Norwegian friends and psychological integration, which may act as a form of validity check.

### Conclusion

Our findings suggest that participants in the current study consider some formal help-seeking sources, such as the GP and psychologist/psychiatrist, for symptoms of depression. However, our findings also suggest that certain informal sources, such as one's partner and Allah/God, may be preferred. Given that help-seeking sources are not mutually exclusive, it is likely that individuals would seek help, or advise someone else to seek help, from a combination of both formal and informal help-seeking sources. However, most participants indicated an average of two barriers to seeking help from the GP. These included, for example, language barriers as well as feeling that the GP would not be able to help. This suggests important areas for interventions and future study. Our study also shows that social ties with the majority population as well as feelings of connectedness with the host country are correlated with considering seeking MH support from the GP. The current study thereby contributes to our understanding of help seeking as dynamic and contingent on cultural context. In line with calls for more non-confirmatory research, which may facilitate making hypothesis tests more informative (78), we encourage the use of our findings as inspiration and basis for future hypotheses. Measures of integration, particularly social and psychological integration, which acknowledge the consequences of shifting cultural contexts, should be considered in future research. Furthermore, future studies should consider longitudinal designs to examine the development of help-seeking preferences, and/or behaviors, over time. Future studies should also consider gender differences in help-seeking. Finally, given our focus on Syrians with a refugee background and a vignette displaying depression symptoms, future research ought to examine a variety of migrant groups as well as employ vignettes with different mental disorders.

## Data Availability Statement

The raw data supporting the conclusions of this article will be made available by the authors, without undue reservation.

## Ethics Statement

The studies involving human participants were reviewed and approved by the Norwegian Center for Research Data. The patients/participants provided their written informed consent to participate in this study.

## Author Contributions

SH contributed to the conceptualization of the study, development of the methodology, conducted the formal analysis, wrote the initial draft, visualization of the findings, and writing- reviewing and editing subsequent drafts. GS contributed to supervision, conceptualization of the study, investigation and data collection, writing- reviewing and editing the manuscript, and acquired funding. HB contributed to the conceptualization of the study, the development of methodology, conducted the formal analysis, investigation and data collection, and writing- reviewing and editing the manuscript. P-EB contributed to supervision, conceptualization of the study, and writing- reviewing and editing the manuscript. LP contributed to the conceptualization of the study and writing- reviewing and editing the manuscript. All authors contributed to the article and approved the submitted version.

## Funding

This project was funded by the Norwegian Research Council (Project Number: 273645).

## Conflict of Interest

The authors declare that the research was conducted in the absence of any commercial or financial relationships that could be construed as a potential conflict of interest.

## Publisher's Note

All claims expressed in this article are solely those of the authors and do not necessarily represent those of their affiliated organizations, or those of the publisher, the editors and the reviewers. Any product that may be evaluated in this article, or claim that may be made by its manufacturer, is not guaranteed or endorsed by the publisher.

## Footnotes

^1^The adult education programs are part of a broader service offered to migrants by the Norwegian authorities and provide training for individuals to qualify for further study or employment in Norway. This includes the introductory program, which is mandatory for refugees, as well as other courses to improve individuals' job and further education opportunities.

^2^Additional analyses suggest that mean HSCL score was negatively correlated with seeking help from, for example, one's partner and mother.
