# Supplementary material for: Integration Is Correlated With Mental Health Help-Seeking From the General Practitioner: Syrian Refugees' Preferences and Perceived Barriers
Source: Front Public Health. 2021 Nov 30;9:777582. doi: 10.3389/fpubh.2021.777582 (PMC8669439; doi:10.3389/fpubh.2021.777582)
Supplement: Supplementary file 1 [file Table_1.DOCX]

**Table SM1.** Participants’ demographic characteristics (N=92). Note that categories with few participants were combined.

|  | **Total (N=92)** | |
| --- | --- | --- |
|  | ***N(%)*** | |
| Age: |  |  |
| <19 | 2 | (2.2) |
| 20-29 | 34 | (37.0) |
| 30-39 | 33 | (35.9) |
| 40-49 | 13 | (14.1) |
| > 50-59 | 10 | (10.9) |
| In a relationship: |  |  |
| Yes | 59 | (64.1) |
| No | 29 | (31.5) |
| 'Missing' | 4 | (4.3) |
| Children: |  |  |
| Yes | 43 | (46.7) |
| No | 45 | (48.9) |
| 'Missing' | 4 | (4.3) |
| Highest completed education: |  |  |
| None | 5 | (5.4) |
| Elementary school (1-10 years) | 20 | (21.7) |
| Highschool (1-5+ years) | 13 | (14.1) |
| University/College (1-5+ years incl. PhD) | 47 | (51.1) |
| ‘Missing’ | 3 | (3.3) |
| Urbanicity: |  |  |
| Big city | 46 | (0.5) |
| Suburbs of a big city | 7 | (7.6) |
| Small to medium town | 21 | (22.8) |
| Village | 9 | (9.8) |
| Sparsely populated area | 5 | (5.4) |
| 'Missing' | 4 | (4.3) |
| Household income (Norwegian kroner): |  |  |
| <150.000 | 23 | (25.0) |
| 150-249.999 | 31 | (33.7) |
| 250-349.999 | 14 | (15.2) |
| 350-449.999 | 8 | (8.7) |
| >450.000 | 12 | (13.0) |
| 'Missing' | 4 | (4.3) |
| Occupation (last 4 weeks): |  |  |
| In paid work (or temporarily absent) | 20 | (21.7) |
| In education (not paid by the employer, or temporarily absent) | 20 | (21.7) |
| Unemployed and actively seeking employment | 17 | (18.5) |
| Unemployed, not active job seeker/permanently ill or disabled | 11 | (12.0) |
| Homemaker/carer | 3 | (3.3) |
| Other | 15 | (16.3) |
| 'Missing' | 6 | (6.5) |

**SM2**. Vignettes presented to participants in original Norwegian language and English translation.

Original:
Karam/Ghazal er en 27-år gammel servitør på en restaurant i Bergen. Han/hun er født i Oslo hvor foreldrene var innehavere av en restaurant. Han/hun har nå bodd i Bergen i 5 år. De siste ukene har han/hun følt seg trist hver dag. Karam/Ghazals tristhet har vært uavbrutt og han/hun kan ikke finne noen forklaring på den ut ifra ting som har skjedd eller årstiden. Det er vanskelig for ham/henne å gå på jobb hver dag; han/hun pleide å trives med kollegaene sine og med arbeidet i restauranten, men nå kan han/hun ikke lenger finne noe glede i det. Faktisk er Karam/Ghazal lite interessert i de fleste aktivitetene som han/hun pleide å like tidligere. Karam/Ghazal er ikke samboende eller gift og bor i nærheten av sin bror/søster. Vanligvis liker de å gå ut sammen og med venner, men nå finner han/hun ikke glede i dette lenger. Karam/Ghazal har veldig dårlig samvittighet fordi han/hun er så trist og han/hun føler at han/hun har sviktet broren/søsteren og vennene sine. Han/hun har prøvd å endre sine arbeidsrutiner og få nye hobbyer for å bli motivert igjen, men han/hun klarer ikke konsentrere seg om disse gjøremålene. Til og med broren/søsteren har nå sagt at Karam/Ghazal blir altfor lett distrahert og at han/hun er ute av stand til å ta avgjørelser. Siden disse problemene begynte, har han/hun sovet dårlig hver natt, han/hun har hatt vanskeligheter med å sovne og våkner mange ganger i løpet av natten. Da han/hun lå våken for noen netter siden og prøvde å få sove, begynte han/hun å gråte fordi han/hun følte seg så hjelpeløs.

English translation:

Karam/Ghazal is a 27-year-old waiter at a restaurant in Bergen. He/she was born in Oslo, where his/her parents owned a restaurant. He/she has now lived in Bergen for 5 years. For the past few weeks, he/she has been feeling sad every day. Karam/Ghazal's sadness has been uninterrupted, and he/she can find no explanation for it based on things that have happened or the time of the year. It is difficult for him/her to go to work every day; he/she used to enjoy spending time with his/her colleagues, and the work in the restaurant, but now he/she can no longer find any joy in it. In fact, Karam/Ghazal has little interest in most of the activities he/she used to enjoy. Karam/Ghazal is not living with a partner or married and lives near his/her brother/sister. Usually, they like to go out together and with friends, but now he/she does not find pleasure in this anymore either. Karam/Ghazal has a very bad conscience because he/she is so sad and feels that he/she has failed his/her brother/sister and his/her friends. He/she has tried to change his/her work routines and start new hobbies to improve his/her motivation again, but he/she is unable to concentrate on these tasks. Even his/her brother/sister has now said that Karam/Ghazal is too easily distracted and that he/she is too indecisive. Since these problems began, he/she has slept poorly every night, he/she has had difficulty falling asleep and wakes up several times throughout the night. When he/she lay awake a few nights ago trying to fall asleep, he/she began to cry because he/she felt so helpless.
